# Effect of fat volume fraction, sodium caseinate, and starch on the optimization of the sensory properties of frankfurter sausages

**DOI:** 10.1002/fsn3.6

**Published:** 2013-01-08

**Authors:** Dimitris Petridis, Christos Ritzoulis, Iakovos Tzivanos, Eleuterios Vlazakis, Emmanuel Derlikis, Vareltzis Patroklos

**Affiliations:** Department of Food Technology, ATEI of ThessalonikiPO Box 141, 57400, Thessaloniki, Greece

**Keywords:** Frankfurter, mixture components, principal component analysis, product optimization, redundancy analysis, sensory, sodium caseinate, starch

## Abstract

The effect of two important nonmeat constituents (starch and sodium caseinate) and fat content on the sensory perception of frankfurter sausages has been assessed for two mixture amounts (17% and 27%). A strong correlation among objective fattiness, elasticity, and chewiness has been established; these correlate negatively to consistency and hardness. This has been attributed to the protein gel disruption arising from local phase separations. Hedonic consistency, elasticity, and chewiness showed a very strong positive correlation to one another. Contour plots, based on responses of principal component axes, show that lard is important in increasing the objective sensory intensities of fattiness, chewiness, and elasticity, and for decreasing hardness and consistency. In higher lard proportions, caseinate and starch decrease the red color intensity and the acceptability of chewiness, elasticity, and consistency. Optimization of the component amounts was performed using response trace plots. After redundancy analysis, sensory and instrumental variables were found in very good mutual agreement; hardness was assessed as the most important mechanical variable, followed by chewiness.

## Practical Applications

A streamlined statistical procedure, known as redundancy analysis, was developed and applied to evaluate and combine mechanical and sensory sets of data regarding different sausage compositions. This work also describes the performance of sensory evaluation using principal component axes, thereby extracting the major information of sensory attributes. The principal axes are then used as response variables into a three mixture components design as to describe optimal product relationships. This procedure helps to identify mechanical and sensory profiles with an emphasis on the practical investigation of the optimal product composition for the consumer market.

## Introduction

During meat processing, a number of exogenous nonmeat substances are commonly added to the product in order to prevent drip loss, increase the product shelf life, and enhance its sensory acceptability (Joly and Anderstein 2009). The latter is related to a number of factors such as appearance, taste, odor, and kinesthetic/mechanical behavior. To this end, substances as diverse as starch and other polysaccharides, proteins and their derivatives, spices, seasonings and flavors, antioxidants, and preservatives can be incorporated into meat products, each fulfilling a specific role in the final product (Cassens 1994; Joly and Anderstein 2009; Sebranek 2009; Xiong 2009).

Macromolecules such as starch are common ingredients in sausages, offering a diverse array of functional characteristics (e.g., Ho et al. 1995; Beggs et al. 1997; Bloukas et al. 1997; Hughers et al. 1997; Lyons et al. 1999; Pietrasik and Duda 2000; Yang et al. 2001). Starch forms a complex with meat proteins when the product is heated to a temperature above the gelation temperature of the starch, at which point the starch swells. Meat proteins form a complex with gelatinized starch granules. The degree of gel strength of the complex is greater than that of meat proteins alone, and the emulsion stability of the proteins is improved (Petridis et al. 2010). Berry (1994) reported improved tenderness in low-fat (8%) pork nuggets by using modified potato or tapioca starches. Kao and Lin (2006) showed that increasing starch level in reduced fat frankfurters resulted in lower G′ (storage modulus) and G′ (loss modulus) of konjac–potato starch mixtures leading to more elastic-mixed gels. No differences in textural hardness among gel-added treatments were noticed.

Milk proteins can also be an important factor in modifying the texture and cooking behavior of sausages. For example, caseinate is reported to be an adequate substitute for meat in frankfurter sausages (Atughonu et al. 1998), acting as surface-active material at the interface between fat globule and meat protein (Su et al. 2000). *β*-Lactoglobulin-enriched milk whey protein fractions can reduce cook loss and increase texture profile analysis (TPA)-monitored hardness, while decreasing the relevant springiness in frankfurters (Hayes et al. 2005). A major challenge in the meat industry is the control of meat swelling under water, especially in the presence of salts (Wilding et al. 1986). Caseinate is a known water-binding material which can contribute toward that aspect in processed meat products (Tsai et al. 1998; Pietrasik and Jarmoluk 2003). Sodium caseinate binds fat and water, thereby increasing yield and reducing shrinkage, while contributing high-quality protein. Although highly effective, sodium caseinate is a milk-based binder and its pricing structure can fluctuate unreliably.

The correlation between fat content and sausage sensory stimulus has been the subject of thorough investigations in the past. Humans are highly sensitive to even slight alterations in the fat content of a sausage (Ritzoulis et al. 2010). Decrease of the fat content in frankfurters has been reported to increase the perceived juiciness of the product, and this has been attributed to the substitution of fat with water in low-fat sausages (Matulis et al. 1995). Frankfurters made with oil-in-water emulsions have been reported to exhibit higher (*P* < 0.05) hardness, springiness, and chewiness values compared with typical samples (Delgado-Pando et al. 2010). On the other hand, reduction of fat has been reported to result in decreased emulsion stability (Crehan et al. 2000).

To assess the sausage texture, TPA has been widely used as an instrumental method, providing information on both the deformation and fracture properties of sausage under large strains (Beilken et al. 1991; Bruna et al. 2000; García et al. 2002; Cáceres et al. 2004). Textural attributes in TPA are often defined from the force–time curves including hardness, springiness, cohesiveness, chewiness, etc. On the other hand, in the statistical analysis, when a process includes many variables, principal component analysis (PCA) is usually used to reduce the number of variables by combining or expressing two or more variables with a single factor. In addition, PCA is used to detect the structure in the relationships between variables (Rahman and Al-Farsi 2005). Some previous studies on food texture tended to apply PCA to reduce the numbers of textural attributes; examples include Toda et al. (1971), Ordóñez et al. (2001), Rahman and Al-Farsi (2005), Dong et al. (2007), and Probola and Zander (2007). As conclusions of these researches, two or three principal components are considered enough to extract a great part of the total variation, and PCA proved to be a good statistical method in reducing and explaining textural factors.

Petridis et al. (2010) have shown that caseinate (where used), starch, and total fat content are all highly important components in a typical sausage formulation regarding sausage texture. Some of the major challenges in the sausage industry involve the determination of the optimum amounts of such substances. Fine tuning of the manufacturing recipe for each product is an essential prerequisite for texture optimization, ingredient economy, as well as for obvious health and environmental implications arising from the excessive use of additives.

This work studies the effect of starch, sodium caseinate, and fat in a typical sausage formulation, as well as the mechanical and sensory properties of the end product. The primary aim is to asses in detail the relative contribution of each one of the three parameters (starch, sodium caseinate, and fat content) to the sensory perception (visual and oral) of a frankfurter sausage. However, instrumental measurements are often not sufficient to render the information provided by sensory evaluation due to two main reasons: (a) the available instruments in some cases are less sensitive than the human senses and (b) in most cases the relationship between instrumental and sensory data is not straightforward (Sieffermann 2005). Therefore, an effort was made to correlate the objective data (instrumental data and trained panelists) to consumer perception (hedonic response of nontrained panelists). This could provide useful data to the meat-processing industry regarding the effect of each one of the three individual ingredients, as well as of the interactions between them. This can also be viewed as an in-depth case study pertaining to the evaluation of the most important sensory and mechanical attributes of frankfurter sausages by elaborating some advanced statistical techniques (principal component and redundancy analysis). Finally, the consumer-optimized composition of the added caseinate, starch, and fat can be used to formulate a marketable product.

## Materials and Methods

### Materials

Selected fat-free porcine and bovine chuck meat, as well as lard, were purchased from a local retail shop. Sodium caseinate (protein ≥88%; ash ≤4.5%; moisture ≤6.5%) was provided from MEVGAL dairies SA (Thessaloniki, Greece). Potato starch (AVEBE UA, Veendam, the Netherlands), phosphate salts (Chemische Fabrik Budenheim KG, Budenheim, Germany), and ascorbic acid (Hebei R&M Healthcare Company Limited, Shijiazhuang, China) were used for the preparation of sausages. Edible skin (gut collagen) was manufactured by NIPPI, Inc. (Tokyo, Japan).

### Sausage preparation

The ingredients used for each batch of sausages were as follows: 1 kg beef, 1 kg pork, 86 g NaCl, 0.3 g sodium nitrite, 2 g ascorbic acid, 0.4 g phosphate, 1000 g ice, lard, sodium caseinate, and starch as per Table 1.

**Table 1:** Design points and combined proportions of components totaling to 17% and 27% mixture amounts.

*n*	Points	Caseinate (%)	Starch (%)	Lard (%)	Mixture amount
1	Vertex	0	0	17	17
2	Vertex	3.1	0	13.9	17
3	Vertex	0	3.1	13.9	17
4	Vertex	3.1	3.1	10.7	17
5	Middle edge	0	1.6	15.4	17
6	Middle edge	1.6	0	15.4	17
7	Middle edge	3.1	1.6	12.3	17
8	Middle edge	1.6	3.1	12.3	17
9	Center	1.6	1.6	13.9	17
10	Axial	0.8	0.8	15.4	17
11	Axial	2.4	0.8	13.9	17
12	Axial	0.8	2.4	13.9	17
13	Axial	2.4	2.4	12.3	17
1	Vertex	0	0	27	27
2	Vertex	5	0	22	27
3	Vertex	0	5	22	27
4	Vertex	5	5	17	27
5	Middle edge	0	2.5	24.5	27
6	Middle edge	2.5	0	24.5	27
7	Middle edge	5	2.5	19.5	27
8	Middle edge	2.5	5	19.5	27
9	Center	2.5	2.5	22	27
10	Axial	1.3	1.3	24.5	27
11	Axial	3.8	1.3	22	27
12	Axial	1.3	3.8	22	27
13	Axial	3.8	3.8	19.5	27

The ingredients were placed in a cutter (Kramer & Grebe, Wallau, Germany) in the following sequence: meat, salt, nitrites, phosphates, and ice were mixed with a program of 12 rpm for 5 min (breaking up stage) at below 0°C; this was followed by the addition of starch, lard, and sodium caseinate at 15 rpm (emulsification stage) at 8–9°C; ascorbic acid was added at 25 rpm (agitation stage) at 12–16°C. The paste was then transferred into a filling machine (Vemag, Robot 500, Verden, Germany), where the edible skin was filled with the paste, forming sausages 15 cm in length. The filled sausages were knotted at the ends, then transferred into a programmable oven, where they underwent a thermal process program as follows: 45°C for 25 min (curing stage), 50°C for 25 min (drying stage), 65°C for 30 min (quick drying), heating to a core temperature of 75°C with 5 min holding time at that temperature, and finally showering in situ for chilling (4°C). They were then removed to dry and were finally stored at 4°C. The core temperature was constantly monitored with a probe inserted in one of the sausages of each lot. Before organoleptic assessment, each sausage was heated for 3 min in boiling water. All sensory tests were repeated at least three times, while all instrumental tests were repeated at least six times.

### Experimental design

The following steps were taken for the buildup of the experimental design and the treatment of the data:

- Choice of a three components mixture design augmented with new points along the sides of the triangle and inside the confined area.
- A specific sensory plan, adapted to the requirements of the mixture design, was selected to obtain sufficient evaluation of sensory attributes.
- The most important mechanical variables that substantially induce the response of the sensory attributes were quantified by means of redundancy analysis.
- The gathered sensory data were treated statistically by first performing PCA in order to extract the first two major axes. These principal axes were then used as responses of the sensory profile, in order to define model equations that provide the best fits by using the three mixture components as independent variables, and thereafter contour and trace plots as product optimization techniques.

**Figure 1:**
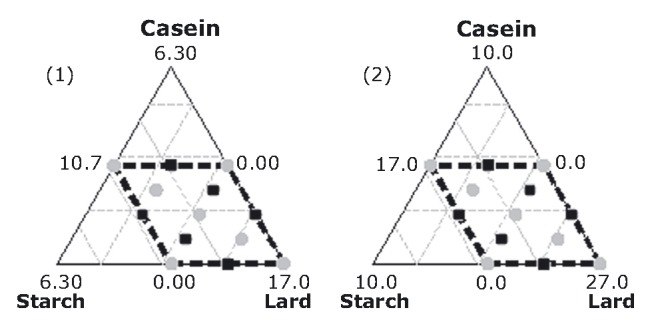
Mixture-amounts design showing the operating area of the triangles and the points of component combinations. Squared points indicate mutual relocation between the two amount designs for the sensory performance.

A mixture experiment with extreme vertices was set up as to conform to the constrained ranges adopted for the three components under study: starch, sodium caseinate, and lard. The lard proportion ranged between 10% and 27% and the other two between 0% and 5%. The lard proportion was judged as being too wide; thus, a mixture-amounts design was adopted, in which the lard proportion was split into two ranges 10–17% and 17–27%, according to the scheme shown in Figure 1 and the proportion combinations in Table 1. This design includes 13 sampling units per mixture amount. Plan 13.25, as obtained from Cochran and Cox (1957), was deemed as the most suitable experimental design. This plan includes *t* = 13 treatments, *k* = 3 treatments per panelist, *b* = 13 panelists, *r* = 5 replicates per treatment, and *λ* = 1 pair of similar treatments per panelist. All of the middle edge and two axial points interchanged between the two mixture-amount designs in the sensory plan, in order to ensure equal contribution of the two lard proportions to the panelists (square points in Fig. 1).

Sensory evaluation based on *objective* perception (i.e., absolute hardness) was performed twice. A panel of 10 members of the School's staff, 30–50 years of age, plus three research students, was selected on the basis of frequent consumption meat products, interest, and availability. The panel members were given a brief initial training using various samples of different sausage formulations with characteristic texture: for example, very elastic and very hard, every time they were asked to participate in the sensory testing. They were trained to assess the following textural attributes:

- Hardness as the force required to penetrate sample with molar teeth.
- Consistency as the evaluation of the amount of deformation before rupture.
- Elasticity as the degree of bouncing between two consecutive bites.
- Fatness as fatty feeling in the mouth and gum.
- Chewiness as the amount of effort that goes into preparing the sample for swallowing.
- Red color intensity as the result of comparison of the degree of redness between slices of the various samples under white light.

### Instrumental design

**Figure 2:**
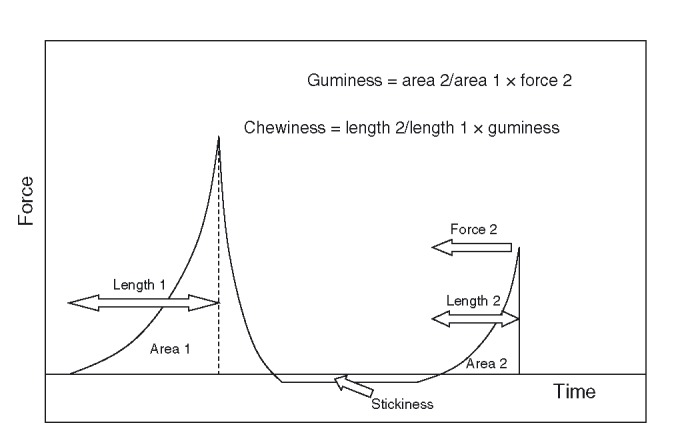
Definition of the various texture characteristics determined by texture profile analysis (TPA).

TPA (Friedman et al. 1963; Bourne 1978) is a well-established method of texture analysis, frequently referred to in the literature as a standard method for texture characterization (Chen 2009). TPA has been found to provide good correlations to sensory analysis for meat products such as rib steaks (Caine et al. 2003) and frankfurter-type sausages (Yang et al. 2001; Ritzoulis et al. 2010). The principle of the method is the application of two successive compressions to a test sample using a mechanical testing machine in imitation of a chewing process. The obtained force–displacement/time curves can be used for an approximate quantification of a number of kinesthetic parameters such as cohesiveness, viscosity, elasticity, adhesiveness, brittleness, chewiness, and gumminess (Fig. 2). Experiments were performed using a TA-XT texture analyzer (TA instruments, New Castle, DE), as described before by Ritzoulis et al. (2010). The analyzer was equipped with a 50-mm-diameter aluminum cylinder, operating with a compression rate of 5 mm/sec. Samples, 20 mm in length, were cut using a dedicated template ring, and axially compressed to 40% of their original height. The capacity of the load cell used was 30 g. All tests were performed at least six times. The OriginPro 8.0 (OriginLab Corporation, Northampton, MA) computer program was chosen to obtain graphic displays of the texture analyzer data and perform the necessary calculations.

Colorimetric tests were performed using a Micro Color LMC tristimulus colorimeter (Dr. Lange, Berlin, Germany). The value of *α* in a Hunter (*L*, *a*, *b*) scale was used as a direct measure of red color intensity. All color measurements were performed at least six times.

### Sensory analysis

Each slice was cut into 3-cm cylinders. Samples were assessed in duplicate at room temperature and were presented to panelists hosted in special booths in white plastic dishes. The order of assessment was randomized within each session. The samples were taken out from refrigeration (4°C) 30 min prior to their testing and left for equilibration at room temperature and were immersed in boiling water for 3 min immediately prior to testing.

The technique used for the sensory assessment was that of the unstructured/universal scaling (Munoz and Civille 1998): the panelists were asked to record their evaluation by drawing a vertical line for each sample across a horizontal line 15 cm long at the point that best reflected their perception of the magnitude of each attribute. The left end (0 cm) of the line was marked as not at all hard to bite, elastic, fatty, texturally consistent, chewy, and reddish. The right end (15 cm) was marked for the texture as very hard to bite, elastic, fatty, texturally consistent, chewy, and reddish.

Potential outliers were detected through a dotplot construction between objective sensory scores and treatments. At each treatment, five replicates were checked for remote values from the rest of the aggregated data.

Sensory evaluation based on *hedonic* perception (i.e., acceptability of an attribute) was run five times using 65 students chosen randomly from the first two semesters thus assuring no previous training experience on sensory tests. Panelists, who were first familiarized with the definition of each attribute as it was predesignated, were asked to rate the *acceptability* of hardness, fattiness, elasticity, consistency, red color, and chewiness of the samples. The same 15-cm unstructured scale line was also applied to the hedonic variables (from “not at all acceptable” to “very acceptable”) in order to be consistent with the objective scaling for statistical comparisons. Low-salt water and unsalted crackers were provided to clean the palate between samples in both objective and hedonic tests.

Following a previously established methodology (Petridis et al. 2010; Ritzoulis et al. 2010), this work combines objective and hedonic assessment of the same attributes (elasticity, cohesiveness, red color, etc.). This aids in establishing a direct link between the absolute value of an attribute (a quantity that can be also linked to instrumental measurements) and the extent of its acceptance. The same attributes are measured using instrumental methods (TPA analysis and colorimetry). The importance of this approach lies in the fact that the effect of a particular component (e.g., fat content) can be directly linked to a sensory attribute (e.g., liking toward elasticity). Thus, the liking of specific hedonic attributes which correspond to the objective and mechanical variables was chosen to produce comparative statistical results which are very important for product optimization.

Adjusted sensory mean scores of the objective and hedonic variables were deduced from the 13 samples of each run.

### Data analysis

Data were statistically treated with the following techniques:

1. A PCA for sensory variables (objective and hedonic) was performed separately for each sensory variable set, as to estimate the loading factors (correlation coefficients) between each variable and the two major axes: the higher the coefficient of that variable, the greater the importance of the component formation. The two major axes were then regressed individually against the mixture components, applying the special cubic multiple regression equation:
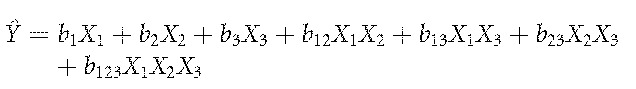
(1)
  Coefficients were calculated for centered amount values (22 = (17 + 27)/2).Backward elimination of variables was followed in order to remove nonsignificant terms, apart from the three main mixture components which were always forced to remain in the final equation form (Piepel and Cornell 1994). It was also assumed that the effect of amount on the dependent variable *Y* was linear; thus, first-order interactions between mixture components and amount were considered in the equation model.A regression model was considered reliable only when the lack of model fit test was not significant (*P* > 0.05) accompanied by high *R*^2^ values of the determined and predicted coefficients. Two replicates for the objective variables and five for the hedonic ones were extracted from the sensory experiments. Contour and trace plots were chosen as to elucidate the mixture optimization conditions derived from the said equation.
2. A redundancy analysis as described by Ter Braak and Wiertz (1994) and performed by the CANOCO statistical software (Ter Braak and Smilauer 2002) was employed in order to find relationships between the objective sensory variables and the mechanical ones. In this analysis, the set of sensory variables expressed in the plot defined by axes 1 and 2 of the PCA is taken as dependent and is regressed against the independent mechanical set.

## Results and Discussion

### Relationship between instrumental texture analysis and objective sensory evaluation

In this study, sensory properties of formulated sausages were evaluated in three ways (a) by instrumental means (absolute values), (b) a trained sensory panel (objective values), and (c) by an untrained sensory panel (hedonic values) to imitate consumer's perception. However, one must be careful when using instrumental data to imitate oral processes (Szczesniak and Hall 1975; Rosenthal 1999). Oral processing is a complicated time-dependent process (Hitchings and Lillford 1988), and therefore, sensory testing has been performed in all formulations. Both objective (i.e., absolute hardness) and hedonic tests (i.e., acceptability of hardness) were performed for all mechanical attributes, in order to obtain data on the objective magnitude of each parameter in relation to its consumer acceptance. Therefore, it was necessary first to establish the relationship between the absolute values obtained by instrumental analysis and the objective values obtained by the trained panelists. This was done by performing a redundancy analysis of variables which has been successfully used in food products by our team in an attempt to quantify relationships between the sensory and mechanical profiles (Raphaelides et al. 1995, 1998). By changing the proportions of some basic ingredients of the product, the texture profile is expected to alter, ultimately affecting the sensory attributes of food. In that sense, the redundancy analysis aims to locate and describe the predominant mechanical variables which control the response of the sensory attributes.

Overall dependent sensory and instrumental variables explained 89.7% of the total variation, regarding the first two major axes. Three criteria of variable importance for the redundancy analysis were chosen: forward selection of mechanical variables, *t*-values of the regression coefficients, and intersect correlation coefficients of mechanical variables with axes 1 and 2 (Ter Braak and Smilauer 2002). Hardness was assessed as the most important mechanical variable (*R*^2^ = 0.48, *t* = 3.19, *r* = 0.544 with axis 1), followed by chewiness (*R*^2^ = 0.28, *t* = 1.86, *r* = 0.237 with axis 2).

**Figure 3:**
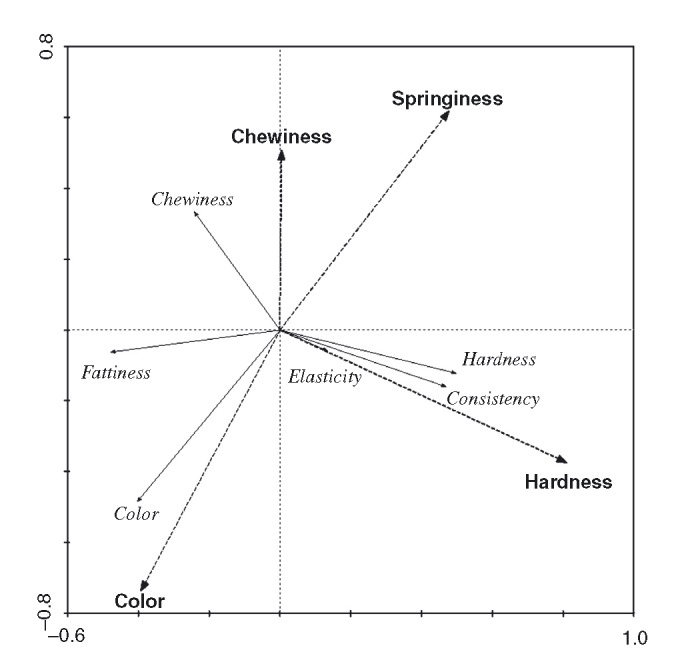
Biplot based on redundancy analysis of sensory profile (solid lines) with respect to instrumental variables (dashed lines). The lines display the approximate correlation coefficients between the two sets of variables. Longer arrows are more important in producing effects.

Potential relationships between the two sets of variables are shown in Figure 3: Mechanical hardness correlates strongly in a positive manner with objective sensory hardness and consistency and negatively with objective chewiness and fattiness. Mechanical chewiness correlates fairly well in a positive direction with objective sensory chewiness, while both color measurement methods (sensory and colorimetric) correlate very strongly and positively to each other.

### Principal component analysis

**Figure 4:**
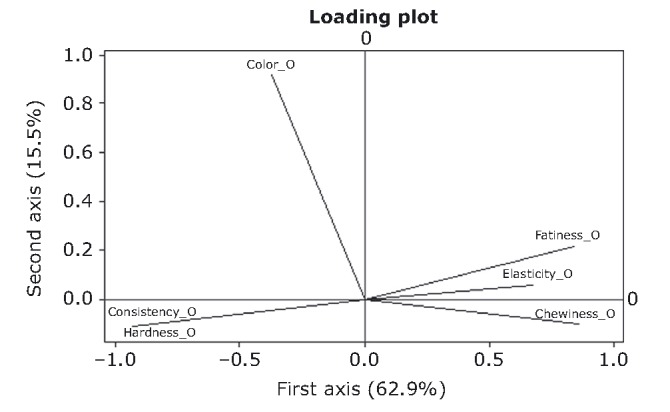
Loading plot of objective sensory variables by the principal component analysis. Arrows indicate the strength of each variable importance and the number in brackets the percentage contribution of each axis to the total variation.

**Figure 5:**
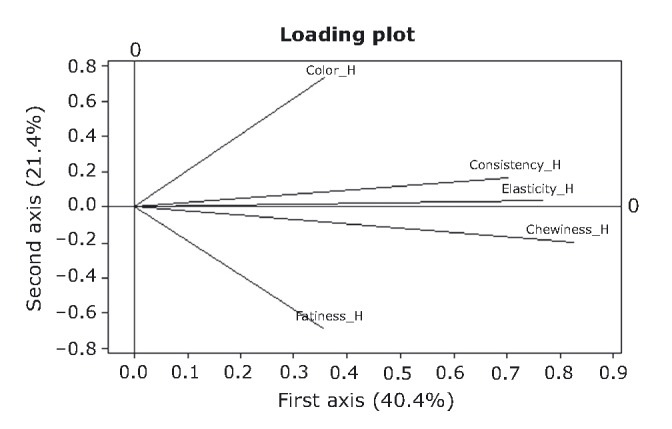
Loading plot of hedonic sensory variables by the principal component analysis. Arrows indicate the strength of each variable importance and the number in brackets the percentage contribution of each axis to the total variation.

PCA was performed on objective and hedonic data in order to distinguish the most important variables (Sharma 1996). Results (Figs. 4, 5) indicate that the first two principal component axes extracted 78.4% and 61.8% of the total variation for the objective and hedonic variables, respectively. The right half of the horizontal axis (axis 1) is explicitly described by a first group of variables, namely objective fattiness, elasticity, and chewiness, whereas a second group, objective consistency and hardness, describe the left half. In Figure 4, arrows forming small oblique angles indicate highly positive correlation coefficients, while obtuse angles indicate highly negative relationships. Loading values (Table 2) suggest that both groups of variables correlate very strongly between variables and with axis 1.

**Table 2:** Loading factors (correlations) between sensory variables and principal component axes and correspondent values between principal component analysis (PCA) scores and unstructured sensory scale.

Variables	Correlation		Rank scaling							
	AXIS 1	AXIS 2	Low (3–6 cm)		Moderate (6–9 cm)		Adequate (9–12 cm)		Fair (12–15 cm)	
Sensory objective										
Fattiness	0.84		−2.2	−0.6	−0.6	1.2	1.2	2.2	>2.2	
Chewiness	0.85		−2.2	−0.5	−0.5	0.8	0.8	2.2		
Elasticity	0.67		−2.2	−0.9	−0.9	0.9	0.9	2.2		
Consistency	−0.93		2.2	0.7	0.7	0.0	0.0	−1.0	−1.0	−2.2
Hardness	−0.93		2.2	1.1	1.1	0.0	0.0	−1.2	−1.2	−2.2
Color		0.92	−2.2	−1.1	−1.1	1.0	1.0	1.8		
Sensory hedonic										
Chewiness	0.83		−2.4	−1.2	−1.2	0.4	0.4	1.8	1.8	2.2
Consistency	0.70		−2.4	−1.4	−1.4	0.9	0.9	2.2		
Elasticity	0.77		−2.4	−1.0	−1.0	0.4	0.4	2.0	2.0	2.2

Taking into account the results from PCA, it is possible to treat all five variables of axis 1, as one. The new variable is now axis 1, which reflects responses of elasticity, chewiness, fattiness, consistency, and hardness. High values of axis 1 correspond to high values of fattiness, elasticity, and chewiness, and low values of consistency and hardness and vice versa. The above are in very good agreement with previous findings where increases in biting force and hardness were associated with decreases in elasticity and chewiness (Petridis et al. 2010). This was attributed to the disruption of the protein gel due to local phase separations between starch and caseinate and/or meat protein. This is further reinforced by observations in caseinate–myofibrillar protein in meat products (Su et al. 2000; Barbut 2007), which suggest local phase separations between the two components. Objective red color appears to be the unique and strong positive contributor for the formation of axis 2 (*r* = 0.92).

PCA on hedonic data showed that consistency, elasticity, and chewiness correlate strongly and positively with each other (Fig. 5) and also with axis 1 (Table 2). High scores of axis 1 correspond to high values of those variables. The above correlation is readily explicable, as elasticity and chewiness are expected to be closely correlated by definition (Szczesniak and Hall 1975; Bourne 1978). The second axis is formed by the hedonic variables red color and fattiness rather loosely. It should be pointed out that, according to the above, *objective* consistency correlates negatively to elasticity and chewiness, whereas *hedonic* consistency correlates positively to the same attributes. This is a valuable indication of the necessity for the differentiation between the objective magnitude of an attribute (i.e., directly comparable to instrumental texture analysis) and its hedonic counterpart.

### Analysis of the effects of mixture components on objective and hedonic sensory properties

Mixture experiments are performed in many product-development designs (Piepel and Cornell 1994). Two or more ingredients (components) are mixed in various proportions, and many attributes, sensory and/or mechanical, of the resulting products are recorded. The measured attributes (responses) can depend either on the proportion of components present in the mixture or on the total amount of the mixture.

The effect of the independent variables, caseinate (X1), starch (X2), and lard content (X3) was analyzed by regression using equation (1). Instead of regressing the independent variables for each separate dependent variable (objective and/or hedonic red color, hardness, consistency, fattiness, chewiness, and elasticity), regression was performed for the four axes (AXIS1_O, AXIS1_H, AXIS2_O, and AXIS2_H). According to PCA results, Axis1_O includes the objective attributes hardness, consistency, fattiness, chewiness, and elasticity, and Axis2_O the objective red color attribute, while Axis1_H and Axis2_H the corresponding hedonic variables.

The response of objective variables (AXIS1_O) is best described by the three components effects and the interaction term starch × lard:


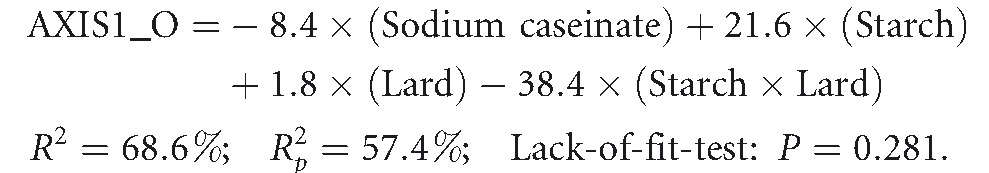
(2)

The different mixture amounts did not affect the panelists' sensory stimuli, but starch and lard show negative synergy (negative sign in the term).

The mixture amounts are very effective in describing the effect of the response objective “red color intensity” (AXIS2_O), alone or combined with interaction terms:


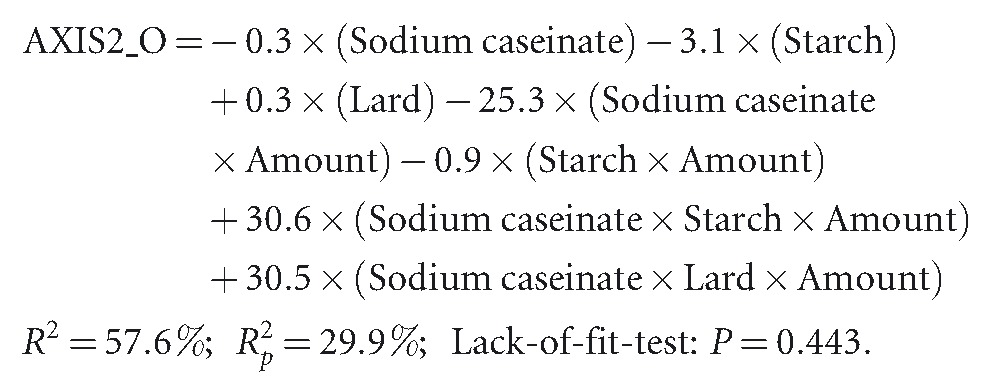
(3)

In higher lard proportions (amount 27%), sodium caseinate and starch decrease the red color intensity (negative signs in the terms), but sodium caseinate, when combined with starch and lard in higher lard amounts, increase the red color intensity. These results should be interpreted with caution, however, as the gap between predicted and determined *R*^2^ values is large (29.9% and 57.6%, respectively).

Mixture amounts are also important for the hedonic variables (AXIS1_H):


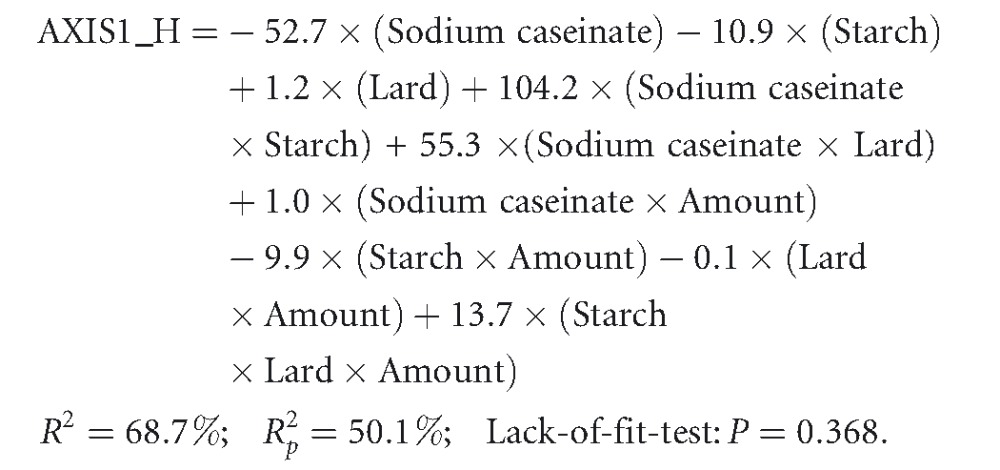
(4)

Starch or sodium caseinate reduces the acceptability of the hedonic variables. Sodium caseinate, when combined with the other components in higher lard proportions, increases the acceptability of the variables under study.

Acceptability toward red color (AXIS2_H) was rejected, despite providing good fit with the mixture components, due to the discrepancy between the predicted and determined *R*^2^ values (10.0% and 43.1%, respectively, results are not shown).

**Figure 6:**
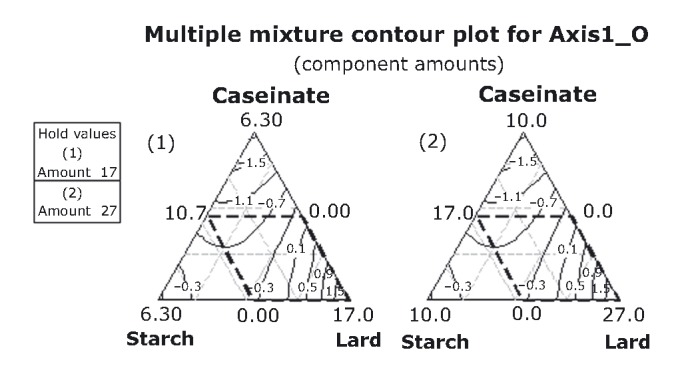
Contour plots for the objective variables at two mixture-amounts designs.

**Figure 7:**
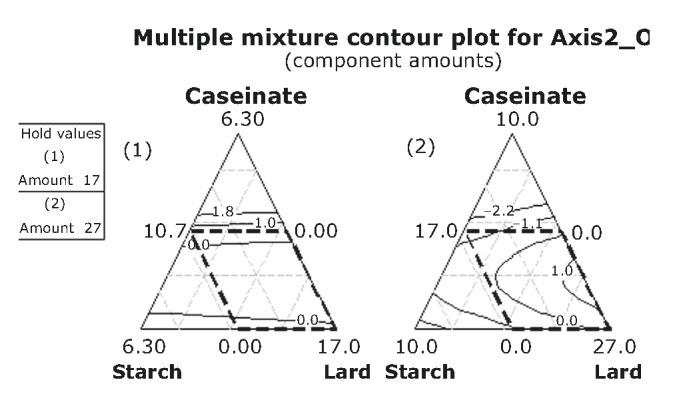
Contour plots for the red color intensity at two mixture-amounts designs.

**Figure 8:**
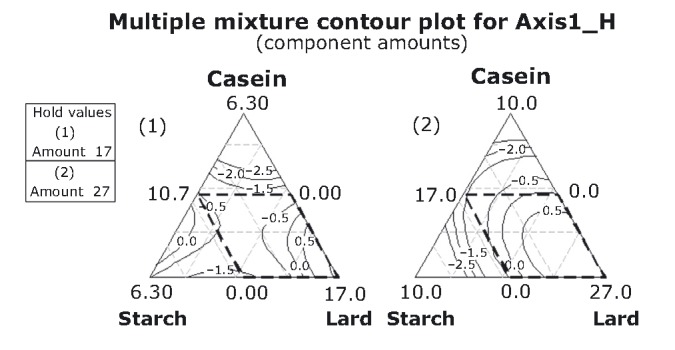
Contour plots for the hedonic variables at two mixture-amounts designs.

In order to optimize the caseinate, starch, and lard content for consumer acceptance, it was necessary to arrange the PCA scores of the objective and hedonic sensory properties to the unstructured scale (0–15 cm) used for the respective sensory properties (Table 2). Results can be better visualized with the contour plots in Figures 6-8. These plots show how a response variable relates to the three ingredients, based on a model equation. Lard is very important for increasing the sensory intensity of fattiness, chewiness, and elasticity, and for decreasing the intensity of hardness and consistency (AXIS1_O), the latter two reaching maximum intensities at very high caseinate and starch proportions (5%) (Fig. 6 and Table 2). Increasing caseinate and starch content was expected to increase consistency. According to Su et al. (2000), the fat globules of a frankfurter batter are confined locally within the denser nonmeat (caseinate) protein matrix. This means that the chances for fat coalescence during cooking may be reduced so that emulsions with high fat and water-binding properties are formed. Thus, the products with firmer texture are expected. As the term “amount” was not found statistically significant for AXIS1_O (eq. (2)), the two mixture designs appear identical in Figure 6 after eliminating the mixture-amount effect.

It should be noted that the contours are fairly symmetrical perpendicular to the lard vertex, indicating a fairly linear effect of the lard component. Deviations from linearity are due to the incorporation of the second-order term starch × lard in the equation (2). This suggests that, practically for all fat contents, the two macromolecular groups act in the same way toward fattiness, chewiness, elasticity, hardness, and consistency. This is in complete agreement with our findings derived from a different experimental (semiqualitative) design (Petridis et al. 2010), implying that both starch and caseinate interact similarly with the myofibrillar matrix of the sausage. Reduction in hardness should be related to phase separation between starch/caseinate and the myofibrillar protein.

Starch and pork meat protein appear not to mutually interact with each other upon heating in temperatures similar to the ones applied in the present experiments (Li and Yeh 2002). It is also reported that increased starch content reduces the elastic modulus of mixed starch–whey protein isolate gels due to a weak starch matrix formation between the two components (Aguilera and Rojas 1996). Such phase separations are normally concentration dependent. Modified starch enhances water binding (Ruusunen et al. 2003), thus increasing the polymers effective concentration. The rheology of whey protein–starch systems shows concentration-dependent transitions from solid like to liquid like (Vu Dang et al. 2009). In these cases, protein and starch appear to phase separate, with one phase dispersing into the other, reducing its elastic modulus. One can argue that starch phase separates and interferes with the continuous meat protein gel, reducing elasticity and its related parameters such as chewiness.

According to the rescaled values in Table 2, fattiness could be characterized in the mixture samples as “moderately intense,” chewiness and elasticity as “moderately to fairly intense,” and finally consistency and hardness as “moderately to fairly intense,” in opposite direction of the former variables (due to the negative signs of the scores).

Red color is important only in the higher proportion amounts (Fig. 7), showing an adequate intensity level at a composition of 2.5% caseinate, 0% starch, and 24.5% lard.

Adequate acceptability toward chewiness, consistency, and elasticity rises, as it reaches high lard proportions in the higher mixture-amounts design (Fig. 8). Moderate levels of acceptability are encountered mostly in the lower mixture-amounts design.

### Optimization procedure

**Figure 9:**
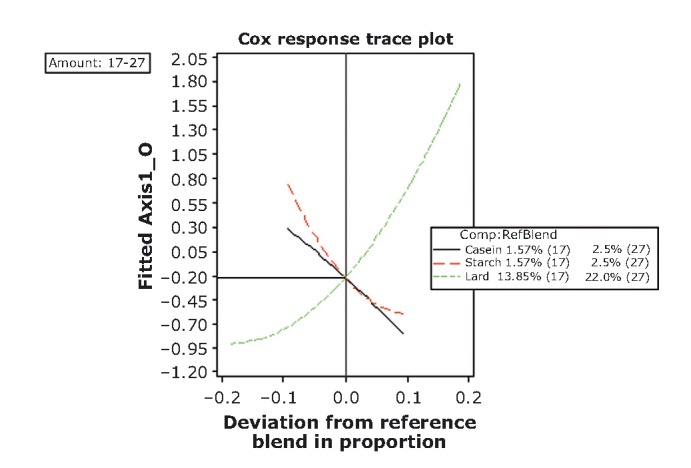
Response trace plots for objective variables including both the mixture-amount designs. As the proportion of lard in the mixture increases (and the other mixture component decrease), the intensity rating of AXIS1_O increases.

**Figure 10:**
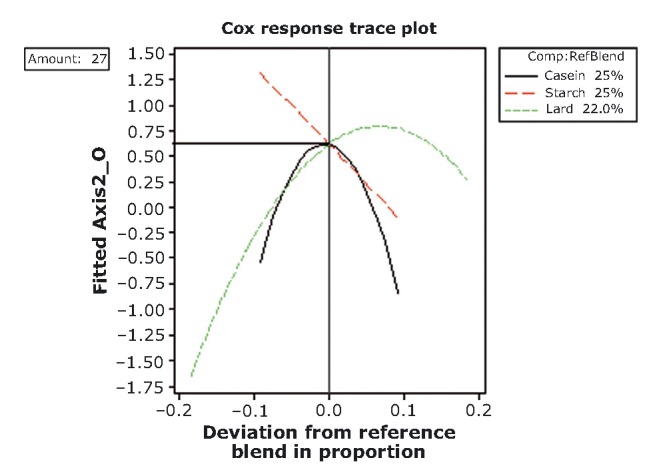
Response trace plots for the red color intensity at the higher mixture-amount designs. As the proportion of caseinate in the mixture increases (and the other mixture component decrease), the rating of red color intensity decreases.

**Figure 11:**
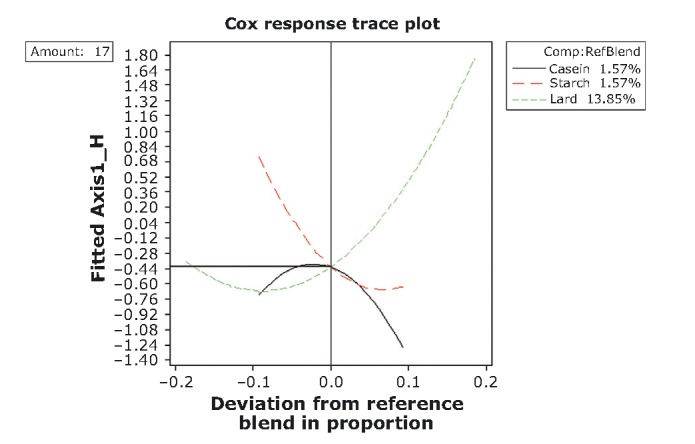
Response trace plots for hedonic variables at the lower mixture-amount design. As the proportion of lard in the mixture increases (and the other mixture component decrease), the acceptance rating of AXIS1_H increases.

**Figure 12:**
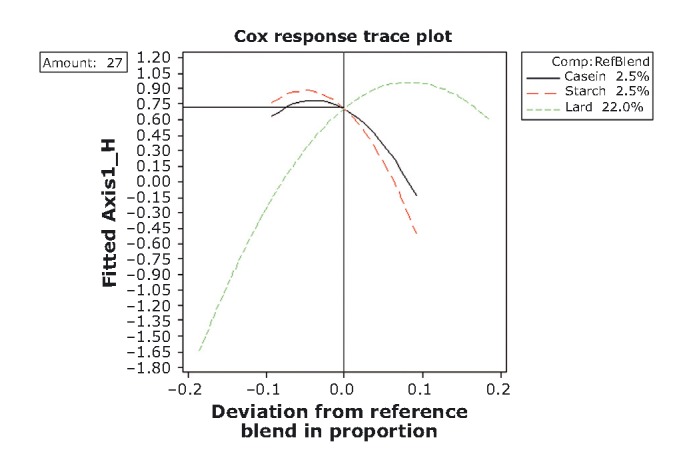
Response trace plots for hedonic variables at the higher mixture-amount design. As the proportion of caseinate or starch in the mixture increases (and the other mixture component decrease), the acceptance rating of hedonic variables decreases.

The ensuing step in our analysis has been the construction of response trace plots as to allow conditions for optimization. Trace or component effects plots show how each ingredient affects the response relative to a reference blend. The center point has been selected as the reference blend (Table 1; Figs. 1 and 9-12). At this point, for both mixture amounts (17% and 27%), the objective variables fattiness, chewiness, and elasticity have reached a moderate range of intensity (−0.20; Table 2 and Fig. 9), whereas the consistency and hardness have already switched to “adequate.” Lard is the most important component, that is, due to its large blend proportion range and distance of response change (extending its influence along the whole scale of both axes in the graph). Thus, increases in the lard proportion lead to an increase in the sensory intensity of score values for fattiness, chewiness, and elasticity, whereas a lard proportion of less than 22.0% or 13.89% (for each of the two mixture amounts) increases the hardness and consistency. This is in good agreement with previous data which suggest that objective sensory attributes such as elasticity and cohesiveness of frankfurters rises monotonically with fat content (Ritzoulis et al. 2010). The importance of the other two components is minor, because the range of reference blend proportion and *Y*-axis distance changes is fairly small.

The intensity of red color reaches the upper moderate level at a response value of 0.60 and only for the higher mixture-amounts design (Fig. 10), as the lower one (17%) was found not to have significant effects (Fig. 7). The red color intensity is reduced linearly with starch addition. In order to achieve optimal red color intensity, caseinate has to reach its maximum level (2.5%) and lard should be close to the maximum level (22%). Dependence of the red color intensity with caseinate has been previously reported by this group (Petridis et al. 2010), where caseinate was found to render the product more opaque, in a manner roughly comparable to that reported by Liu et al. (2007), attributed to the lowering of *L*-values in breakfast pork sausages due to water removal and subsequent reduction of the diffused color. Caseinate is a known water-binding material for meat products (Tsai et al. 1998; Pietrasik and Jarmoluk 2003). It can bind water from the gel matrix, reducing light diffraction, hence lowering the *L*-value of the sausages. As far as the effect of lard toward red color intensity is concerned, Pietrasik (1999) reports that redness values a* were inversely proportional to fat content, due to the increase of yellow-hue components of lard. The lower fat levels in the work in question coincide with the intermediate-to-high fat levels in the present experiment. In the overlap region between the two works, the objective red perception does indeed reduces with the increase in fat.

The center point for the acceptability of variables at the lower mixture amount (lard + caseinate + starch = 17%) corresponds to the moderate range of chewiness, consistency, and elasticity (response value −0.44, Fig. 11). Lard is again the most important component. At lower amounts of fat (negative deviation from the central point), the acceptability levels are low and remain so up to a content of levels of 13.85%. From that point onward, the acceptability increase is monotonic with lard content, approaching eventually maximal acceptable scores at the maximum amount of lard 17% corresponding to the range of “adequate.” Close to the center point caseinate proportion, 1.57% has reached its maximum acceptable score while starch acceptable scores decrease up to fairly high concentrations.

A different behavior is observed for the higher mixture amount (lard + caseinate + starch = 27%) (Fig. 12). At the reference blend point, all the component proportions (2.5 – 2.5 – 22%) approach their maximal acceptable scores which correspond to the range of “adequate.” Further increases in caseinate and starch proportion reduce the acceptability. It is noteworthy that caseinate and starch act in practically the same way toward acceptability.

## Conclusions

A statistical technique, known as redundancy analysis, proved to be an efficient validating tool for the correlation between objective sensory and mechanical attributes and is therefore potentially used for product sensory analysis.

The response of objective and hedonic variables is well described by the three mixture components: lard, starch, and sodium caseinate. The results can be better visualized in contour and trace plots after correlating the extracted principal component scores with particular ranges of values of the initial unstructured scale used. Under this approach, lard is found to be of high importance in increasing the objective sensory intensity of fattiness, chewiness, and elasticity, in decreasing the intensity of hardness and consistency and finally, in increasing the hedonic chewiness consistency and elasticity. Sodium caseinate and starch, in the higher lard amounts, decrease the intensity of red color. For the higher mixture amount (lard + sodium caseinate + starch = 27%), high contents of sodium caseinate and starch reduce the sensory acceptability, an important finding when considering the manufacture of sausages using relatively high amounts of nonmeat materials. The above could lead to a marketable product.
